# Retraction: Hypersexuality Addiction and Withdrawal: Phenomenology, Neurogenetics and Epigenetics

**DOI:** 10.7759/cureus.r1

**Published:** 2015-10-06

**Authors:** Kenneth Blum, Rajendra D Badgaiyan, Mark S Gold

**Affiliations:** 1 Department of Psychiatry, McKnight Brain Institute , University of Florida,; 2 Department of Psychiatry, and Laboratory of Advanced Radiochemistry, University of Minnesota ,School of Medicine, Minneapolis , MN., USA; 3 Departments of Psychiatry & Behavioral Sciences, Keck School of Medicine of USC, Los Angeles, CA, USA

This article has been retracted by agreement between all of the authors, John R. Adler (Cureus Editor-in-Chief) and Cureus. The retraction has been agreed upon due to erroneous statements about the existence of sexual addiction and hypersexuality codes within the Diagnostic and Statistical Manual of Mental Disorders, Fifth Edition (DSM-5). In an effort to unambiguously rectify all errors, the authors have agreed to submit a significantly revised manuscript for subsequent peer review and re-publication.

